# Social Competence Intervention for Parents (SCI-P): Comparing Outcomes for a Parent Education Program Targeting Adolescents with ASD

**DOI:** 10.1155/2012/681465

**Published:** 2012-04-18

**Authors:** Tia R. Schultz, Janine P. Stichter, Melissa J. Herzog, Stephanie D. McGhee, Kristin Lierheimer

**Affiliations:** ^1^Department of Special Education, University of Missouri, Columbia, MO 65211, USA; ^2^FPG Child Development Institute, The University of North Carolina at Chapel Hill, Chapel Hill, NC 27599, USA

## Abstract

Research has shown that parent education programs can address some of the distinct challenges that parents of youth with autism spectrum disorders (ASDs) encounter. This study examined the effectiveness of the Social Competence Intervention for Parents (SCI-P), a parent education program, administered in conjunction with a social competence intervention that targeted youth with ASD ages 11–14 (SCI-A). Using a quasi-experimental pre-post design, parents were assigned to either the SCI-P group (*n* = 16) or to the waitlist comparison group (*n* = 10). Analyses of covariance (ANCOVAs) revealed a significant effect for parent education participation such that SCI-P participants experienced significantly greater reductions in levels of stress and a trend for increases in parenting sense of competence from pre- to post-intervention. Moreover, parents in the SCI-P group reported high satisfaction with the program. These findings suggest that parent education can result in positive outcomes for parents' well being.

## 1. Introduction

The variety of skill deficits displayed by youth with autism spectrum disorders (ASDs) present a unique set of challenges to their parents [1, 2]. Studies indicate that parents who experience these challenges report increased stress levels and a diminished sense of competence as a parent, which have been associated with feelings of social isolation, increased risk for clinical depression, and reduced marital satisfaction [3–5]. Potentially exacerbating these negative outcomes is ongoing difficulties, as reported by parents, in accessing information related to their child's diagnosis [6]. Parent education programs are designed to provide parents with information to increase their knowledge [7], and as a result, have the potential to decrease stress [8] and increase parents' sense of competence [9].

### 1.1. Parent Stress

The often unpredictable and somewhat ambiguous social and behavioral challenges presented by children with ASD can be a primary source of stress for parents. For example, literature on parents of children with ASD consistently indicates that these parents report higher levels of stress than their peers who are parenting typically developing children [10, 11]. Additionally, there is consistent evidence that the stress experienced by parents of children with ASD is greater than that experienced by parents of children with other types of developmental disabilities [12–14]. These parents also report a decreased level of social support, which has been linked to increased stress and poor adaptation to parenting a child with ASD [3]. Further, research has shown a link between high parent stress and reduced effectiveness of child intervention [15, 16].

Addressing stress in parents of children with ASD is imperative given the potential for negative outcomes for parents, children, and family interaction dynamics. Much of the research related to effective parenting in general has shown that parents who experience high levels of stress are less responsive and effective parents for their children [17]. Additionally, interpersonal factors in parenting dyads, such as conflict (e.g., over how to handle children's behavioral problems) and low perceived levels of support from one's partner, have been associated with parental distress that can spillover into parent-child interactions [18]. Parental support and education may be beneficial in providing parents of children with ASD with information that will aid them in effectively managing their caregiver roles and the stress associated with these roles. In turn, increased success as primary caregivers enhances the likelihood for decreased anxiety, risk of parental depression, and family disruption.

### 1.2. Parenting Sense of Competence

Another factor important to effectively manage parenting roles, and hence essential when working to improve parental outcomes in families with children with ASD, is parental sense of competence. Parenting self-competence is a multidimensional construct that incorporates many elements, including feeling satisfied with and effective in the parenting role [19, 20]. Extant research has primarily focused on parenting self-efficacy or a parent's sense of their ability to effectively parent their child [17]. Hastings and Brown [21] identified maternal self-efficacy as meditating the association between problematic child behavior and mothers' mental health. According to Gilmore and Cuskelly [19], maternal self-efficacy may provide mothers with increased resilience to both anxiety and depression. Sanders and Woolley [14] suggest that parent-training programs should include elements aimed at increasing parents' perceptions of self-efficacy. More specifically, mothers who receive educational materials and assistance in dealing with problematic child behavior report feeling more competent as parents [9]. Although researchers and educators can rarely control parents' use of the information they receive, the existing literature suggests that simply having access to the information is associated with positive outcomes for parents. Unfortunately, starting at the initial diagnosis, many parents have difficulty accessing this information [6, 22].

### 1.3. The Need for Parent Education for Parents of Youth with ASD

Previous literature has indicated that parents of children with ASD place a high priority on receiving information about their children's disability [23, 24]. Moreover, they often request access to education as a way of gaining information about developments in the field of autism, and practical information, such as how to cope with behavioral problems [25]. Parent education programs may be an effective mechanism, not only for providing information, but also for reducing parent stress and increasing parent sense of competence. For example, one study found that parents who participated in an educational program that provided them with information related to their child's autism diagnosis reported decreased parenting stress and increased parenting self-efficacy [26]. In contrast, parents participating in a parent support group specifically targeting decreases in parent stress and increases in self-efficacy only reported mild changes in these domains [27]. Given parents' reports that information on ASD and related interventions are their most pressing need [25], improved parent outcomes when such information is provided are not surprising [26].

Accessing meaningful and accurate information is compounded for parents as their children enter adolescence. Youth with autism spectrum disorders face unique social and developmental challenges compared to their typically developing peers. The need to access meaningful and accurate information is heightened for parents as their children enter adolescence, when the lack or misuse of social skills is often magnified (e.g., the increasing social demands of school, less tolerant peers). These difficulties, if untreated, can limit postschool outcomes and adult relationships (see [28] for a review). Parents of these individuals are concerned about both these current behaviors and long-term outcomes. Thus, providing parents with information on what is typical for children, what they can do to help improve their child's skills, and what other parents in similar situations are experiencing has the potential to greatly impact parents' stress and their sense of competence as a parent.

 Recently, research has begun to evaluate parent-assisted social skills intervention for youth with ASD. Researchers [29, 30] evaluated the Children's Friendship Training (CFT) program, which involved teaching youth (both elementary and adolescent) skills for developing and maintaining friendships. In CFT, children attended sessions with peers while parents concurrently attended sessions to learn how to implement the training techniques with their children. Positive outcomes were found for the youth's abilities to develop and maintain friendships. However, parent outcomes were not assessed. Although some studies focus on child/adolescent outcomes related to parent education participation [31], others focus on outcomes for parents in particular. Existing research indicates a range of positive outcomes related to parent education participation [32, 33]. For example, Ingersoll and Dvortczak [7] evaluated a program that taught parents naturalistic techniques and strategies for teaching their children social communication skills at home. This program yielded increases in parents' reported knowledge of skills and high levels of parent satisfaction. Keen et al. [26] found that parents who participated in a parent-training program reported reduced stress. Other studies have observed changes in parent affect [34] and parent-child interactions [35] from pre- to postintervention. A recent review of parent education research for parents of children with ASD noted that most studies that have evaluated parent outcomes have measured skill increases, with only a few measuring changes in parent stress [31]. Thus, additional research is needed to assess the impact of parent education on specific indicators of parents' well-being, such as sense of competence as a parent and parent stress.

### 1.4. Current Study

The current study evaluates the effectiveness of the Social Competence Intervention for Parents (SCI-P), a parent education program developed as a supplement to the Social Competence Intervention for Adolescents (SCI-A) with ASD [36]. The SCI-A program targets youth with ASD who display marked deficits in the area of social interactions. Previous research has found positive social and behavioral outcomes for youth who participated in the SCI-A program [36]. In response to parents' requests for information, the SCI-P program was created as a supplement to SCI-A in order to provide parents with important information and strategies related to supporting the social competence development of their child. The primary aim of the current study was to examine the impact of SCI-P participation versus nonparticipation on specific indicators of parents' well-being. In particular, it was hypothesized that, controlling for preintervention assessment scores, parents who participated in SCI-P would report lower stress and higher sense of competence than parents in the waitlist comparison group at the postintervention assessment.

## 2. Methods

### 2.1. Participants and Procedure

Participants in this study were the parents of children who participated in an after-school social competence intervention program for youth (SCI-A) at a Midwestern interdisciplinary autism center. The social skills intervention targeted youth between the ages of 11 and 14 with a documented diagnosis of ASD (e.g., Asperger's Syndrome, Autistic Disorder, or Pervasive Developmental Disorder—Not Otherwise Specified). Additional screening criteria included clinically significant scores on either the Autism Diagnostic Observation Scale (ADOS) or Autism Diagnostic Interview-Revised (ADI-R); moderate-to-high cognitive functioning (full-scale IQ scores at or above 75); and at least one hour daily participation in school- or community-based settings with typically developing same-age peers (e.g., general education classrooms, boy scouts). Youth were provided 20 hours of social competence intervention delivered from a fully developed curriculum, in a group setting that met for an hour, twice a week for 10 consecutive weeks. As noted by Stichter and colleagues [36], SCI-A participants have demonstrated significant gains in pivotal social cognition skills and significant improvements in social interaction, and executive function behaviors were also reported. For a more detailed description of results and an overview of SCI-A, see Stichter et al. [36].

Over the course of four academic semesters, the parents/guardians of the 27 youth enrolled in the SCI-A program were invited to participate in the parent education outcome study. Of those invited, only the parents of one youth declined consent to participate in the current study (96.3% recruitment). Using a quasiexperimental design [37], parents were distributed into the SCI-P group (parents and youth both received intervention) or the waitlist comparison group (only the youth received intervention) based on the timeslot families chose for participation in the youth program (i.e., parents were automatically in the waitlist comparison group if the parent education program did not occur concurrent with their child's intervention time). All parents participating in the current parent outcome study completed a set of self-report assessments approximately two weeks prior to and two weeks after the conclusion of the youth intervention (interval between pre- and postassessments was approximately 14-15 weeks). These parents also completed standardized assessments of their participating child's social behaviors and interactions. All parents, regardless of assignment to the SCI-P or the waitlist comparison group, received a basic status report on their child's progress through the SCI-A curriculum.

To alleviate concerns of nonindependence, data from only one parent per youth were included in the current analysis. Demographic information about parents and youth is reported in Table 1. The SCI-P group included 16 mothers with a mean age of 46.69 years (SD = 7.07). Average age of youth (*n* = 16 boys) was 12.68 years (SD = 1.36). These youth had a mean full-scale IQ score of 97.44 (SD = 14.81) and ranged from 5th to 9th grades. Most of the SCI-P families included two biological or adoptive parents and had a distribution of annual incomes similar to the local population. The waitlist comparison group included eight mothers and two fathers, with a mean age of 41.20 years (SD = 7.90 years). The children (*n* = 10 boys) of the waitlist comparison group parents had a mean age of 12.62 years (SD = 1.13 years), were in the 5th–8th grades, and were of average intelligence (*M*_IQ_ = 102.67, SD = 11.60). Half of the waitlist comparison group families included two biological or adoptive parents and income levels similar to the local population. All parents in both groups reported themselves and their children as White. Independent samples *t*-tests were conducted to determine if the SCI-P and waitlist comparison groups were similar on background characteristics. No significant differences were found on parent age, youth age, youth IQ, or family income.

### 2.2. Parent Education Program

Parents in the SCI-P group attended the parent education program for a total of 20 hours (twice per week for 10 weeks) while their children attended concurrent sessions. There were four distinct parent education groups over the course of the study. SCI-P modal group size was six parents (on occasion two parents per youth attended). The program focused on strategies for parents to teach and support social skill development in their children. The parent sessions were led by a Parent Educator, who was a doctoral student in special education and certified as a family life educator. In addition to having master's level training in parent education, the parent educator also received doctoral level training in special education and on-going supervision by doctoral level professionals in the field. Topics covered were the same as those evidenced as effective in the SCI-A program (greetings/eye contact/acknowledging others, facial expressions, sharing ideas, turn taking in conversation, feelings/emotions, and problem solving).

The teaching methods utilized were derived directly from the research on effective instruction in parent education groups (e.g., [7, 38, 39]). Each unit involved didactic instruction of the new skill and time for planning how and when to target the skills at home. Skills were verbally explained, modeled, and practiced with feedback from the parent educator during each unit. There were also voluntary homework assignments in each unit for parents to do with their children at home. The homework included an example activity for targeting the skills taught in the unit. Parents were allowed to individualize the homework activities to match their family needs. Homework was turned into the parent educator who provided feedback to the parents in a subsequent session.

The process used for developing the parent curriculum involved several steps. First, the first author met with the second author, a professor in special education who has expertise in curriculum design and who was responsible for the content and structure of the youth program curriculum. Together they reviewed key constructs to link each unit of the youth program to the parent program. They adapted the material from the youth program to be appropriate for a parent education program based on the research of effective parent programs. Next, the first author compiled all the information for the second author to review before delivery. Finally, the second author provided ongoing consultation during delivery.

Fidelity data on implementation process and content were collected on 50% of all sessions for the parent education groups. Fidelity data were collected live during the representative SCI-P sessions by master's and doctoral level special education students by assigning one point for each accurately completed behavior or activity on a predesigned fidelity checklist. Process fidelity consisted of elements related to the way the program was delivered (e.g., previous material was reviewed, skills were modeled, and parents had time to ask questions). Fidelity percentages were calculated by adding the number of fidelity points earned by the number of points available. Mean process fidelity was 87 percent. Content fidelity consisted of concepts that needed to be covered each lesson (e.g., concept definitions). Mean content fidelity was 94 percent. Interrater reliability data were collected for more than 35% of all sessions. The percentages of interobserver agreement (IOA = agreements/sum of agreements and disagreements) averaged 95.5%.

Of the 16 SCI-P parents in the current study, mean parent attendance was 77.5% of the parent education sessions (SD = 18.9%, range = 25%–100%). Most parents attended 15 sessions or more and modal attendance was 95% of sessions (*n* = 4). It should be noted that although one mother with poor attendance (25%) was the pre/post data respondent, her husband was the primary SCI-P attendant. It is unknown to what degree this couple shared SCI-P intervention outside of the group setting. There was also a homework assignment each week for parents to do with their children at home. Homework was turned into the parent educator who provided feedback to the parents. An average of 32.7% (SD = 31.3%, range = 0%–100%) of these voluntary homework assignments were completed and returned. Despite not completing the formal homework sheet, many parents shared examples of using techniques and trying activities at home. In particular, parents described creating games for their child to practice concepts learned in sessions and creating visual aids to support their child's use of techniques at home. Parents who expressed the greatest difficulty in completing homework were those who indicated having specific additional stressors at home (e.g., a partner who was ill, a demanding work schedule, or being the only parent in the household).

### 2.3. Measures

Pre- and postmeasures were scheduled and administered by a member of the research team in the absence of the parent educator, at a time and place different than parent education sessions. Parents did not receive any incentives for completing the measures. Parents in both the SCI-P and waitlist comparison groups completed the same measures approximately two weeks before the SCI-A program began and two weeks after SCI-A ended.

#### 2.3.1. Parenting Stress

Using a 5-point Likert-type scale, parents reported perceptions of their own stress on the Stress Index for Parents of Adolescents (SIPA; [40]). The adolescent domain (40 items) measured stress levels related to the target youth's characteristics (moodiness, social isolation/withdrawal, delinquent/antisocial behavior, failure to achieve/persevere). The parent domain (34 items) measured stress levels related to the effect of parenting on other life roles (life restrictions, relationship with spouse/partner, social alienation, and feelings of guilt). Scores were summed within each subscale, and subscales were summed to form domain scores. Higher scores indicate greater perceived stress levels for that domain. Using a sample of nearly 1000 parents of children with numerous disorders characterized in part by challenges in social competence, scale developers reported high internal consistency for the SIPA domains and total score (*αs* > .90) and high test-retest reliability coefficients (*rs* > .87; [36]).

#### 2.3.2. Parenting Sense of Competence

The Parenting Sense of Competence scale (PSOC; [41]) assessed feelings of competence as a parent and feelings of comfort and value in that role. The efficacy subscale (eight items; e.g., “I meet my own personal expectations for expertise in caring for my child”) assessed parents' perceptions of their own skills and knowledge related to being a good parent. The Satisfaction subscale (nine items; e.g., “Being a good parent is a reward in itself”) assessed parents' affective perceptions about their value of and comfort in the parenting role. Responses were scored on a 6-point Likert-type scale. Items within each subscale were summed and higher scores indicated greater feelings of Efficacy or Satisfaction in the parental role, respectively. Moderate internal consistency has been reported (Efficacy, *α* = .80; Satisfaction, *α* = .69; [37]).

#### 2.3.3. Youth Social Skills

All parents completed the Social Responsiveness Scale (SRS; [42]) as a pre- and postassessment of their child's social skills, behaviors, and characteristics. The SRS is a standardized 65-item rating scale that measures social impairments on a 4-point Likert-type scale. The SRS generates both a raw score and a norm-referenced *t*-score; for research purposes the raw score is utilized to decrease concerns of ceiling effects. The total raw score is reported in the current study, and higher scores indicated greater social impairment.

#### 2.3.4. Social Validity

At the end of the intervention, parents in the SCI-P group completed a social validity assessment that was adapted from Wheeler et al. [43]. The assessment aimed to capture parents' perceptions of their experiences participating in the parent education program. There were seven statements with responses on a 5-point Likert-type scale (5 =* strong agreement* and 1 =* strong disagreement*). Statements included, “I gained knowledge about social skill development during the parent education program,” and “I have made changes to the way I teach my child new skills.” There were also five open-ended statements that allowed parents to respond with what they liked and what they would have changed about the program.

## 3. Results

As a first step, to indicate the equivalence of the SCI-P and waitlist comparison groups on baseline scores, a series of one-way ANOVAs were conducted. No significant differences were found for preintervention scores on dimensions of parent stress (SIPA), on dimensions of parent sense of competence, or on youth social behavior (SRS; all *P-*values >.27).

### 3.1. Parenting Stress

An analysis of covariance (ANCOVA) was conducted to determine whether the postintervention stress in the Adolescent Domain for the SCI-P and waitlist comparison groups differed after adjustments for preintervention differences. The assumption of homogeneity of regression slopes was confirmed by a nonsignificant interaction between the baseline score and the intervention, *F*(1,21) = 0.02, *P* > .89, and the assumption of homogeneity of variance was confirmed via the Levene test, *F*(1,23) = 3.85, *P* > .06. After adjusting the group means for scores on preintervention Adolescent Domain Stress, the effect of intervention was not significant, *F*(1,22) = 1.36, *P* > .25, partial *ε* ^2^ = .058.  Table 2 provides a descriptive summary of all unadjusted pre- and postintervention scores and the results of the ANCOVAs.

An ANCOVA was conducted to determine whether the postintervention stress in the Parent Domain for the SCI-P and waitlist comparison groups differed after adjustments for preintervention differences. The assumption of homogeneity of regression slopes was confirmed by a nonsignificant interaction between the baseline score and the intervention, *F*(1,21) = 2.21, *P* > .15, and the assumption of homogeneity of variance was confirmed via the Levene test, *F*(1,23) = 0.72, *P* > .40. After adjusting the group means for scores on preintervention Parent Domain Stress, the effect of intervention was significant, *F*(1,22) = 5.47, *P* < .05, partial *ε* ^2^ = .199, indicating that parents in the SCI-P group reported significantly less Parent Domain Stress than their waitlist comparison counterparts.

Given the significance of change in the Parent Domain, exploratory ANCOVAs were conducted to examine if there were specific aspects of stress that were particularly significant. Results indicated that parent intervention status was not a significant predictor of stress about Life Restrictions, *F*(1,23) = 4.17, *P* > .05, partial *ε* ^2^ = .153, or Social Alienation, *F*(1,23) = 0.69, *P* > .41, partial *ε* ^2^ = .029, after controlling for preintervention scores on these subscales. Parent intervention status was a significant predictor of reductions in stress about Parental incompetence, *F*(1,23) = 6.03, *P* > .05, partial *ε* ^2^ = .208, with parents in the SCI-P group evidencing significantly less stress about incompetence than parents in the waitlist comparison group.

Finally, an ANCOVA was conducted to determine whether the postintervention Total Parenting stress for the SCI-P and waitlist comparison groups differed after adjustments for preintervention differences. The assumption of homogeneity of regression slopes was confirmed by a nonsignificant interaction between the baseline score and the intervention, *F*(1,20) = 0.67, *P* > .42, and the assumption of homogeneity of variance was confirmed via the Levene test, *F*(1,22) = 4.38, *P* = .05. After adjusting the group means for scores on preintervention Total parenting stress, the effect of intervention was significant, *F*(1,22) = 4.54, *P* < .05, partial *ε* ^2^ = .178. Parents in the SCI-P group reported significantly less total stress postintervention than their waitlist comparison counterparts.

### 3.2. Parenting Sense of Competence

An ANCOVA was conducted to determine whether the postintervention Sense of Parenting Efficacy for the SCI-P and waitlist comparison groups differed after adjustments for preintervention differences. The assumption of homogeneity of regression slopes was confirmed by a nonsignificant interaction between the baseline score and the intervention, *F*(1,22) = 0.46, *P* > .50, and the assumption of homogeneity of variance was not confirmed via the Levene test, *F*(1,24) = 6.84, *P* < .05. After adjusting the group means for scores on preintervention Sense of Parenting Efficacy, the effect of intervention approached statistical significance, *F*(1,23) = 3.49, *P* = .075, partial *ε* ^2^ = .132. Parents in the SCI-P group had a marginally higher sense of parenting efficacy than their waitlist comparison counterparts.

An ANCOVA was conducted to determine whether the postintervention Sense of Parenting Satisfaction for the SCI-P and waitlist comparison groups differed after adjustments for preintervention differences. The assumption of homogeneity of regression slopes was confirmed by a nonsignificant interaction between the baseline score and the intervention, *F*(1,22) = 0.66, *P* > .42, and the assumption of homogeneity of variance was confirmed via the Levene test, *F*(1,24) = 1.13, *P* > .29. After adjusting the group means for scores on preintervention Parent Domain Stress, the effect of intervention was not significant, *F*(1,23) = 0.45, *P* > .50, partial *ε* ^2^ = .019.

### 3.3. Youth Social Behavior

In addition to the primary focus on parent outcomes, it was necessary to examine the influence of Youth Social Behavior, as indexed by the parent-reported total score on the SRS, given that the youth were participating in the concurrent SCI-A program. First, an ANCOVA was conducted to determine whether the postintervention SRS scores for the children of parents in SCI-P and waitlist comparison groups differed after adjustments for preintervention differences. The assumption of homogeneity of regression slopes was confirmed by a nonsignificant interaction between the baseline score and the intervention, *F*(1,22) = 0.21, *P* > .65, and the assumption of homogeneity of variance was confirmed via the Levene test, *F*(1,24) = 1.02, *P* > .32. After adjusting the group means for scores on preintervention SRS scores, the effect of intervention was not significant, *F*(1,23) = 0.14, *P* > .71, partial *ε* ^2^ = .006. Although the youth's social behavior did not vary across parents' intervention status, paired samples *t*-tests revealed significant improvements in social behavior for youth whose parents were in the waitlist comparison group, *t*(9) = 5.42, *P* < .001, and the SCI-P group, *t*(9) = 4.22, *P* < .01.

 Finally, for the domains on which significant parent intervention group effects were found, it was important to explore the role of youth social behavior. As such, an ANCOVA was conducted to determine whether the postintervention Parent Domain Stress for the SCI-P and waitlist comparison groups differed after adjustments for preintervention differences on Parent Domain Stress and pre- and post-Youth Social Behavior. The assumption of homogeneity of regression slopes was confirmed by a nonsignificant interaction between the pre- and post-SRS scores and the intervention and the assumption of homogeneity of variance was confirmed via the Levene test. After adjusting the group means for scores on preintervention Parent Domain Stress, and pre- and post-youth SRS scores, the effect of parent intervention was significant, *F*(1,20) = 5.34, *P* < .05, partial *ε* ^2^ = .217. Parents in the SCI-P group had significantly less postintervention Parent Domain Stress (adjusted *M* = 67.60, SD = 17.13) than their waitlist comparison counterparts (adjusted *M* = 80.50, SD = 15.64). Similar results were obtained for Total Parenting Stress, *F*(1,19) = 4.66, *P* < .05, partial *ε* ^2^ = .197. Again, parents in the SCI-P group had significantly less postintervention Total Parenting Stress (adjusted *M* = 200.21, SD = 35.61) than their waitlist comparison counterparts (adjusted *M* = 228.10, SD = 44.04).

### 3.4. Social Validity for the SCI-P Program

Table 3 provides the mean scores for each item on the social validity survey provided by parents in the SCI-P group (*n* = 16). All items had mean scores above four on a scale of one to five, indicating that parents agreed with the statements in the survey regarding their satisfaction with the experience and knowledge gained in the SCI-P program.

Parents also responded to open-ended items about their perceptions of the program. In response to the item regarding the style of the program, parents commonly said they liked the ability to discuss and work with other parents. One parent said “…Parents not only learned from the material but from each other as well.” In response to the item regarding things parents liked about the content of the program, parents commonly said they liked learning about how social skills could be broken into small pieces. One parent said “…learning how social interactions take place—all of the different rules and steps. Discovering how intense all of the social rules can be.” In response to the item about parents' views of the most important things learned from participating in the program, parents commonly said they learned how to take their child's perspective. One parent said “How important it is to break every little detail down. I did not realize that before. I also learned to be more positive and approach things with a better attitude.” In response to the item regarding what parents would change, most parents said “nothing.”

## 4. Discussion 

Parent education is an important component of autism intervention, as it can provide positive outcomes for parents and, in turn, their children [32, 33]. This study investigated the impact of parent education participation on parents' stress and sense of competence as a parent. Data indicated that parents who participated in the parent education program (SCI-P) experienced improvements in specific measures of parental well-being, namely, perceived parenting stress, compared to their waitlist comparison counterparts. 

Indications of the stress associated with parenting a child with ASD is a reliable finding in the extant research (e.g., [10, 11]). Consistent with this literature and, as hypothesized, those parents in the SCI-P group reported significantly reduced stress levels on the SIPA total parenting stress measure. Specifically, parents had equivalent levels of stress prior to SCI-P, but at postintervention, parents who participated in SCI-P reported significantly less stress than those parents who were in the waitlist comparison group. Interestingly, the effect of parent intervention status was observed for stress related to the Parent Domain, but not for stress related to the Adolescent Domain. In particular, within the Parent Stress Domain, parents' stress about perceived incompetence was significantly reduced for the SCI-P group. Taken together, these results suggest that parents' *concerns *about their children's behavior were not impacted, but their *own cognitive reactions to these concerns* and their ability to deal with these concerns were reduced. In other words, parents remained concerned about their children but had gained skills related to dealing with those concerns. 

As is common with most parent education research, this study did not control for the degree of use of the educational materials that parents received. However, return of homework and requests to the parent educator during and after sessions for assistance in individualizing the information provided indications of active use. Although this study did not analyze the exact mechanism between information provision, use, and parents' stress, some research suggests that having information about their children, diagnosis-related expectations, and behavioral support strategies are associated with reduced stress (see [31]). Further, parents in the SCI-P group had ongoing access to the parent educator who could answer questions and support parents in working with their children. It may be that this ability to access a qualified professional, if and when parents choose, provides a sense of ease and thus a reduction in stress about one's ability to handle the challenges of parenting. 

Given the provision of information and practice opportunities in the SCI-P group, we hypothesized that these parents would report an increase in their sense of competence. The effect of parent education participation on the sense of parenting self-efficacy approached but did not attain statistical significance. In this study, all parents, regardless of parent intervention status, received progress reports and updates on their child's behavior and progress in the SCI-A program. Although the group setting and ongoing contact with parents and a parent educator may be an important component to feel reduced stress, feelings of competence may be boosted from even minimal educational information and supports [26]. Little is known about the pivotal or optimal levels of intervention necessary for parents to achieve an increase in sense of competence. Parents' reports of satisfaction with being a parent did not differ across parent intervention status. This observation is not surprising given that the SCI-P program did not focus on parents' own enjoyment with being a parent. Analysis of the key components, and more fine-grain outcome measures, would be highly informative for the development of a continuum of effective parent education programs, tailored to specific levels of need. Future research should also explore the parental characteristics related to the needed dosage of parent education for increasing sense of competence. In addition, collecting followup and generalization data on the youth may provide further insight on the potential effects of parent education. 

Given the reciprocal nature of youth social and behavioral concerns and parents' perceptions of their own well-being, it is critical to understand how these factors operate together in the context of intervention. As expected based on prior evidence of the SCI-A program [36], parents' reports of youth social interactions and behaviors improved significantly after youth participated in SCI-A. Interestingly, these youth improvements did not vary across parents' participation in the SCI-P program. This finding is in contrast to the CFT program [29] in which children whose parents attended group parent training yielded positive changes in social skills when compared children whose parents only received materials. However, the parent training sessions in the CFT program focused on learning particular skills for completing specific tasks (such as supporting the child in setting up a play date), whereas the SCI-P program taught parents how to address foundational social competence skills (such as reading a facial expression). The SCI-P program is based on the SCI-A program, which focuses on social constructs and pivotal skills toward reaching social competence. Therefore, the skills parents worked on in SCI-P were designed to enhance the degree to which they were able to support and generalize their child's growth. The degree to which the impact of these parental behaviors had on their children may extend the designated nature of the standardized measures that have traditionally been used to assess the impact on SCI-A. Additional measures would need to explore this potential. Nevertheless, participation in the parent education program did have a unique positive impact on parents' well-being over and above the impact of their child's social and behavioral challenges. 

In the context of the positive outcomes realized for the parents who participated in the parent education group, as with all studies, several limitations should be acknowledged. One limitation of this study is the small sample size, which limits the generalizability of the results. Another limitation is that our design was quasiexperimental by the virtue of assigning parents to parent education based on chosen time slots as opposed to a completely randomized control trial. Although no preintervention differences were detected across groups in the current study, future investigations with additional resources would provide important contributions through the use of randomized control designs. In addition, based on the geographical location of the study, the sample demographics were not highly representative of a diverse population. Further assessment of the impact of parent background characteristics (e.g., level of education) on outcomes such as stress and sense of competence would certainly require investigation across various locations and demographic profiles. Finally, our intervention was only 20 hours and did not include formal childcare for siblings. As a result, although attendance by parents was generally good, it was at times compromised. In most cases, both parents (for those two-parent families) could not attend the SCI-P sessions simultaneously. It is unclear what impact, if any, additional attendance by one or both parents may have had on the current results. 

## 5. Conclusion 

Youth with ASD have a variety of complex social, behavioral, and communicative deficits that impact long-term outcomes for both them and their families [1, 2]. Parents of youth with ASD are at risk for increased stress levels, social isolation, and disrupted family dynamics [3–5]. Additionally, parents of youth with ASD report difficulty in accessing information related to their child's ASD [6], which may be linked to decreased feelings of parenting efficacy. However, prior research has found that parent education programs may support increased parental knowledge [7], be linked to decreased parental stress [8], and may also increase participants' sense of parental competence [9]. 

Results from the current study suggests that participation in a 20-hour parent education program focused on information and strategies related to social competency for youth with ASD may have benefits for parents. Parents who participated in the SCI-P parent education program attributed high levels of social validity (satisfaction with both the content and experience) to the program. Specifically, participants in the SCI-P group reported significantly decreased stress levels from pre- to postintervention, as compared to the parents in the waitlist comparison group. Increases in parents' sense of competence were not impacted by parents' intervention group status. Taken together, these results suggest that parent education is a promising, and likely necessary, component of a comprehensive program of supports for youth with ASD and their families. Further research is needed to investigate which characteristics and amounts of parent education programs are needed in order to maximize the benefits of these programs for parents of youth with ASD. 

## Figures and Tables

**Table 1:** Participant demographic information.

	SCI-P group	Wait list comparison group
	(*n* = 16)	(*n* = 10)
*Parent characteristics*		
Sex		
Mothers	100.0%	80.0%
Fathers	0.0%	20.0%
Age (years)		
*M* (SD)	46.69 (7.07)	41.20 (7.90)
Range	35–58	31–54
Race/ethnicity		
White	93.8%	100.0%
Latino	6.3%	0.0%
Marital status		
Married	81.3%	60.0%
Divorced	12.5%	20.0%
Never married	0.0%	10.0%
Widowed	6.3%	10.0%
*Youth characteristics *		
Sex		
Boys	100.0%	100.0%
Girls	0.0%	0.0%
Age (years)		
*M* (SD)	12.68 (1.36)	12.62 (1.13)
Range	10.92–14.75	11.25–14.25
Race/ethnicity		
White	87.5%	100.0%
Latino	12.5%	0.0%
Full scale IQ		
*M* (SD)	97.44 (14.81)	100.80 (12.43)
Range	77–129	84–130
*Family characteristics*		
Composition		
Two biological parents	50.0%	40.0%
Two adoptive parents	18.8%	10.0%
Stepfamily	12.5%	30.0%
Single parent	18.8%	20.0%
Income		
$10,000–20,000	6.3%	0.0%
$20,000–30,000	6.3%	20.0%
$30,000–40,000	18.8%	10.0%
$40,000 or more	56.3%	40.0%
Did not report	12.5%	30.0%

**Table 2:** Difference in outcomes for SCI-P participants versus waitlist comparison participants.

	SCI-P participants (*n* = 16)				Waitlist comparison participants (*n* = 10)				*F*	*df*
	Pre		Post		Pre		Post			
	*M*	SD	*M*	SD	*M*	SD	*M*	SD		
Parenting stress										
Adolescent domain	112.07	21.71	104.38	21.24	116.20	24.84	113.70	26.53	1.36	1, 22
Parent domain	78.33	14.76	67.60	17.13	78.10	19.90	80.50	15.64	5.47*	1, 22
Total stress	217.86	31.35	199.13	34.57	227.50	40.61	228.10	44.04	4.54*	1, 21
Sense of competence										
Efficacy	27.63	4.26	32.25	4.39	26.50	5.04	28.70	5.58	3.49^+^	1, 23
Satisfaction	39.19	5.14	41.50	5.90	36.60	6.57	38.10	7.28	0.45	1, 23
Youth social behavior	107.00	18.40	90.94	17.26	109.30	22.75	90.60	18.39	0.14	1, 23

Note: Unadjusted pre and post means are reported for all measures. ANCOVA used with baseline scores on each outcome measure used as a covariate.

Note: Youth social behavior measured by parent-reported total score on the Social Responsiveness Scale (SRS).

Note: ^+^ *P* < .10, *P < .05, **= *P* < .01

**Table 3:** SCI-P program social validity scores.

Social validity item	*M*	SD	Range
I gained knowledge about social skill development during the parent education program.	4.63	.50	4-5
I learned a lot about the way I parent during the parent education program.	4.25	.93	2–5
I have made changes to the way I teach my child new skills.	4.38	.72	3–5
My child has shown improvements in his/her social skills.	4.31	.48	4-5
I enjoyed participating in the parent education program.	4.63	.72	3–5
Participating in this program was a good use of my time.	4.67	.82	2–5
I would recommend this program to other parents whose children are participating in the social competence intervention.	4.88	.34	4-5

Note: Response options were 1 (*strongly disagree*) to 5 (*strongly agree*).
